# Optimization of light source parameters for photoacoustic imaging: trade-offs, technologies, and clinical considerations

**DOI:** 10.1088/2515-7647/ae889d

**Published:** 2026-07-21

**Authors:** Kalloor Joseph Francis, David Veysset, Hwidon Lee, Brett E Bouma, Gijs van Soest

**Affiliations:** 1 Department of Cardiology, Thorax Center, Cardiovascular Institute, Erasmus University Medical Center, Rotterdam, The Netherlands; 2 Wellman Center for Photomedicine, Massachusetts General Hospital and Harvard Medical School, Boston, MA, United States of America; 3 Department of Optics and Mechatronics Engineering, Pusan National University, Busan, Republic of Korea; 4 Engineering Research Center for Color-Modulated Extra-Sensory Perception Technology, Pusan National University, Busan, Republic of Korea; 5 Institute for Medical Engineering and Science, Massachusetts Institute of Technology, Cambridge, MA, United States of America; 6 Department of Precision Microsystems Engineering,Faculty of Mechanical Engineering, Delft University of Technology, Delft, The Netherlands

**Keywords:** photoacoustic, optoacoustic, light source selection, signal-to-noise ratio, maximum permissible exposure, clinical translation

## Abstract

The selection of light sources for photoacoustic imaging, a biomedical imaging technology, remains a critical yet under-informed topic. This review discusses the key trade-offs in light source parameters (e.g. wavelength, pulse energy, repetition frequency), safety, and practical considerations. We analyse these factors in the context of underlying imaging physics and the limits imposed by regulatory standards across various wavelength ranges. Attention is given to light source selection for photoacoustic tomography, optical and acoustic resolution microscopy, and endoscopy, which pose different requirements for pulse energies and repetition frequencies, to achieve real-time imaging at the desired depth. Current PA imaging light sources, including high-power lasers with optical parametric oscillators, fibre lasers, laser diodes, vertical-cavity surface-emitting lasers, and light-emitting diodes, are reviewed in terms of wavelength availability, energy, repetition frequency, beam quality, portability, and safety. Clinically viable systems require careful consideration of fibre coupling limitations, maximum permissible exposure at the tissue interface, imaging field of view, and signal-to-noise ratio. Further practical considerations include energy efficiency and thermal management in clinical settings. We also outline the present gaps in the available light source technologies, where the development of new sources may enable new PA imaging capabilities, both scientific and clinical. By offering an overview of these considerations, this review aims to guide researchers, industry, and clinicians in selecting optimal light sources and advancing the development of light sources that address current gaps in available technology.

## 1 Photoacoustic imaging and light sources

Photoacoustic imaging is a technology that allows the creation of volumetric images of optical absorption in opaque media such as biological tissues. Photoacoustic imaging is useful for imaging pathologies that produce changes in optical absorption, such as aberrant vascular patterns associated with specific tumours [1], lipid accumulation in artery walls [2], or increased collagen levels in children with muscular dystrophy [3]. Exogenous contrast agents can be applied to identify disease with a molecular signature, but no innate absorption change [4]. The technology has been reviewed many times, see e.g. [5].

Photoacoustic imaging relies on ultrasonic waves as the information carrier, generated by the thermoelastic effect. Most commonly, a short pulse of light is absorbed, a rapid temperature rise follows, and tissue momentarily expands, creating an instantaneous pressure. Upon relaxation of the pressure, a broadband ultrasonic wave is emitted, which can be recorded using an ultrasound transducer. The recorded waveform encodes where the optical absorption occurred and at what magnitude, modified by acoustic propagation and the detector response. Inversion of the transducer signals is then performed to recover the spatial source pressure distribution, which can then be presented as an acoustic image of absorbed optical energy. Further modelling of optical propagation in the tissue can result in absolute quantification of the optical absorption coefficient, linked to the underlying concentration of absorbers.

Key operating parameters from the light source point of view in photoacoustic signal generation are wavelength and related line width, pulse duration, pulse energy, and repetition frequency. The chosen wavelengths determine biochemical or molecular contrast, as chromophores absorb differently across the spectrum. Especially for endogenous chromophores, there is limited freedom in choosing optimal wavelengths, as the absorption spectra of biological molecules are fixed. Pulse duration determines the acoustic bandwidth and the dimension at which stress confinement holds, defining the resolution of the detected chromophores. Wavelength, pulse energy, and repetition frequency govern the achievable signal within safety limits, and averaging improves the signal-to-noise ratio (SNR) with a sufficiently high frame rate. These constraints make the light source central to a photoacoustic system’s performance and image quality.

Several prior reviews have treated the selection of photoacoustic excitation sources, but mostly within a narrow technology or application scope. For instance, low-cost illumination based on pulsed laser diodes (LDs) and high-power Light-emitting diodes (LEDs) has been surveyed with an emphasis on affordability and system integration [6–9], and portable, diode or LED-driven tomography has been reviewed as a route to point-of-care PA imaging [10, 11]. Beyond these cost-focused sources, modality and spectral-window reviews highlight how imaging geometry and wavelength choice shape system requirements, from the beam quality and pulse parameter constraints of photoacoustic microscopy (PAM) [12] to opportunities and limitations in the wavelength window referred to as short-wave infrared or the second near-infrared window [13, 14]. To these focused reviews, we add a comprehensive, physics-based presentation of light sources, explicitly linking wavelength, pulse duration, pulse energy, and repetition frequency to achievable SNR under maximum permissible exposure (MPE) limits, while also accounting for clinical realities like light delivery, field of view, energy efficiency, and thermal management. We analyse these coupled parameters and discuss their inherent trade-offs across tomography, microscopy, and endoscopy. We compare the practical considerations of major source classes, including optical parametric oscillator (OPO)-based lasers, LDs, VCSELs, LEDs, and fibre sources, with the aim of guiding an informed choice of light source for new systems and applications. Finally, we review gaps and opportunities to guide future source development, combining physical and clinical constraints.

This review examines light sources for biomedical photoacoustic imaging with an emphasis on pulsed optical excitation. Frequency-domain photoacoustics using continuous-wave, modulated, or coded excitation, and alternative excitation modalities including microwave and x-ray illumination, are not covered. We only consider the light source for the excitation of the signal. All-optical implementations of photoacoustics apply acoustic detection by interferometry [15–17], and thus require a light source for readout of the signal. This is usually a frequency-stabilized laser to detect the small modulation of the optical path affected by the passage of the acoustic wave. We will not include these readout lasers. Likewise, we will omit related technologies such as pulsed photothermal optical coherence tomography (OCT), using broadband superluminescent diodes or fast wavelength swept lasers in a phase-sensitive OCT system to detect photothermal expansion [18, 19] and photoacoustic remote sensing [20], which relies on detection of a reflectivity change caused by a pressure-induced refractive index modulation.

### 1.1 History of light source development

Photoacoustic excitation began with intensity modulation, long before the birth of lasers. Alexander Graham Bell’s Photophone in 1880, used sunlight chopped at audio rates to generate sound, proving that modulated light can drive acoustic waves, seeding the concept of photoacoustics [21]. Gas analysis based on the optico-acoustic effect was demonstrated in 1938, showing that broadband sources could produce measurable signals [22]. Early pulsed excitation arrived through electrical means in 1961, where a wire spark produced short optical bursts and generated acoustic signals in solids [23]. The first biomedical laser experiment was conducted in 1964, using a ruby laser that delivered microsecond pulse trains to produce sound from tissue *in vivo* [24]. Laser illuminated spectrophones matured for gas detection using a pulsed ruby laser and chopped CO$ _2$ and HeNe lasers, which provided insights into the SNR gains from narrow line, wavelength selective light and from precise modulation control [25, 26]. In 1973, high-pressure xenon lamps with monochromators introduced photoacoustic spectroscopy [27, 28].

The photoacoustic field widened in scope and scale through the 1980s and 1990s. Microwave pulsing confirmed that thermoelastic generation generalizes beyond light, generated the first thermoelastic signals from *in-vivo* human tissue [29], and enabled 2D imaging using an array detector [30]. The first laser-based photoacoustic images were created using a microscope and a chopped HeNe laser [31]. Multispectral photoacoustic studies emerged from research into ablation of arterial plaques, and showed differential responses of tissue at 248 nm (from a KrF laser), 308 nm (from a XeCl laser), and 532 nm (frequency-doubled Nd:YAG) [32]. Subsequent experiments with a tunable dye laser showed spectral photoacoustic contrast between healthy and diseased arterial tissue. In 1993, a fibre-coupled Q-switched Nd:YAG laser with a 20 ns pulse width and 10 Hz pulse repetition frequency (PRF) generated the first *in-vivo* pulsed PA signals from a human finger (using, coincidentally, a prototype probe for intra-arterial imaging) [33]. An experimental setup using a pulsed Xe-lamp enabled the first PA imaging experiment in the sense that an absorbing object could be located in a large turbid medium, verified with echo [34]. The relation between pulse duration, absorption strength, and the properties of the generated acoustic signal were explored theoretically and experimentally [35, 36], clearly demonstrating that short pulses such as those generated by Q-switched lasers were advantageous. These sources set the template for early photoacoustic tomography, capitalizing on short pulses with high energy.

From the 2000s onward, tunability, high repetition frequency, and portability drove source development. Dye lasers enabled multi-wavelength angiography and functional haemoglobin mapping and seeded optical resolution PAM (OR-PAM) [37–39]. OPO-pumped systems then combined broad tunability with ample pulse energy for three-dimensional small animal imaging and rapid multispectral scans, becoming research workhorses when both energy and wavelength access were needed [40, 41]. Compact, passively Q-switched fibre and microchip lasers raised repetition frequencies and simplified fibre delivery, improving practicality for faster microscopy and integrated systems. Semiconductor emitters shifted the field toward portability. High power pulsed LEDs proved feasible for photoacoustics, then scaled to multiwavelength arrays for bedside and handheld systems while accepting lower pulse energies and leveraging high repetition frequencies for averaging [42, 43]. Pulsed LDs, first confined to 905 nm at pulse energies of tens of $\mu$J and PRFs in the kHz range for two-dimensional point scanning, enabled ultrahigh frame rate imaging near 803 nm when paired with clinical ultrasound receivers, offering new opportunities for pulse energy, repetition frequency, and SNR for ‘real-time’ applications [44, 45]. Most recently, VCSEL arrays enabled thin, addressable patches for three-dimensional haemoglobin mapping and core temperature estimation, pointing toward conformal, efficient, multi-element sources that ease light delivery without fibres [46]. Across these milestones, each technology change shifted what wavelengths, pulse durations, energies, and frame rates were practical under MPE limits, which now defines the optimization space for clinical photoacoustic imaging.

### 1.2 Parameters influencing photoacoustic signal

#### 1.2.1 Photoacoustic signal generation using pulsed light

Under thermal and stress confinement conditions, the initial pressure rise, $p_0$, resulting from the sudden thermal expansion of the light-absorbing chromophores can be simply written as [47]:

\begin{align*} p_0 = \Gamma\,\mu_a\,\Phi,\end{align*}

where $\Gamma$ is the Grüneisen parameter (dimensionless), $\mu_a$ the optical absorption coefficient (m$ ^{-1}$), and $\Phi$ the local fluence [48] (J m$ ^{-2}$), derived from the pulse energy $E$ (J) delivered over an illuminated area $A$ (m$ ^{2}$),

\begin{align*} \Phi = \frac{E}{A}.\end{align*}

The thermal and stress confinement conditions require the laser pulse width, $\tau_\mathrm{p}$, to be much shorter than both the characteristic thermal diffusion time, $\tau_{\mathrm{th}}$, and the stress relaxation time, $\tau_\mathrm{s}$, respectively. They are defined as:

\begin{align*} \begin{aligned} \tau_{\mathrm{th}} &amp; = \frac{d^2}{\alpha_{\mathrm{th}}}, \\ \tau_\mathrm{s} &amp; = \frac{d}{c_\mathrm{s}}, \end{aligned}\end{align*}

where $d$ is the characteristic size of the target chromophore (or feature size), $\alpha_{\mathrm{th}}$ is the thermal diffusivity (m$ ^{2}$ s$ ^{-1}$) of the tissue, and $c_\mathrm{s}$ (m s$ ^{-1}$) is the speed of sound of the medium.

In biological tissues, the stress confinement condition is more stringent, as the thermal diffusion time is typically multiple orders of magnitude longer than the stress relaxation time. For instance, for a feature size of $15\,\mu\mathrm{m}$, the thermal confinement time is $\tau_{\mathrm{th}} \approx 10^{-3}\,\mathrm{s}$, whereas the stress relaxation time is $\tau_\mathrm{s} \approx 10^{-8}\,\mathrm{s}$. Therefore, in practice, single-digit nanosecond ($10^{-9}\,\mathrm{s}$) laser pulses satisfy this condition for feature sizes larger than $15\,\mu\mathrm{m}$, which generate photoacoustic waves possessing a corresponding $-3$ dB acoustic bandwidth of about $50\,\mathrm{MHz}$. The acoustic spectrum is ultimately shaped jointly by the object’s dimensions, which define the intrinsic high-frequency content, and by the laser pulse duration, which introduces an additional low-pass filter with $f_{\max} \approx 1/\tau_\mathrm{p}$. Consequently, when longer pulses are used, the high-frequency components are increasingly suppressed, thereby reducing edge contrast. For these reasons, single-digit nanosecond laser sources are typically preferred in PAI.

For the remainder of this section, we assume thermal and stress confinement conditions in which $p_0$ is proportional to the optical fluence $\Phi$ (equation (1)).

#### 1.2.2 SNR as a function of pulse energy and averaging

The SNR of the measured acoustic wave generated by a single laser pulse is proportional to the initial pressure rise $p_0$ and inversely proportional to the noise $\sigma_\mathrm{p}$ at the detector,

\begin{align*} \mathrm{SNR}_1 \propto \frac{p_0}{\sigma_\mathrm{p}} = \frac{\Gamma\,\mu_a\,\Phi}{\sigma_\mathrm{p}}.\end{align*}

Averaging $N$ independent acquisitions reduces uncorrelated noise by a factor of $\sqrt{N}$, yielding an overall SNR improvement of $\sqrt{N}$,

\begin{align*} \mathrm{SNR}_N \propto \frac{\Gamma\,\mu_a\,\Phi\,\sqrt{N}}{\sigma_\mathrm{p}}.\end{align*}

Assuming a PRF $f$ and a measurement window $T$, the number of averaged pulses is $N = f\,T$. Consequently,

\begin{align*} \mathrm{SNR}_N \propto \Phi\,\sqrt{f}\,\sqrt{T}.\end{align*}

Equation (6) shows that SNR improves linearly with fluence and with the square root of laser repetition frequency and measurement time [49, 50]. This is a major consideration in photoacoustic system design: with a given energy budget under the MPE, averaging high-PRF, low-energy pulses can be less effective than using a single higher-energy pulse. Furthermore, since the SNR increases as $\sqrt{T}$ for a given PRF, averaging quickly becomes impractical unless a high PRF can be achieved. The PRF is, in fact, adjustable for some systems, such as pulsed LDs, but fixed and low (10–100 Hz) for most Q-switched lasers, as detailed in the following sections.

Additionally, the maximum image penetration depth, or the depth from which signals can still be received, sets an upper limit on the repetition frequency to avoid temporal overlap between acoustic waves generated by consecutive laser pulses:

\begin{align*} f < \frac{c_\mathrm{s}}{h}\, .\end{align*}

Here $h$ is the maximum imaging depth that is usually limited by optical attenuation (e.g. approximately $150\,\mathrm{kHz}$ for $h = 1\,\mathrm{cm}$). This constraint is largely relaxed in PAM, where the imaging depth is much shallower, where the Rayleigh length of the focusing optics determines the range of depth sensitivity.

Conversely, the maximum measurement window $T$ is limited by tissue stability, as physiological motion and drifts can corrupt photoacoustic signals over extended integration times, and long acquisition times are impractical in clinical settings. The lower bound of $T$ is the single pulse duration.

#### 1.2.3 Safety-constrained fluence and impact on SNR:

**Figure 1.**
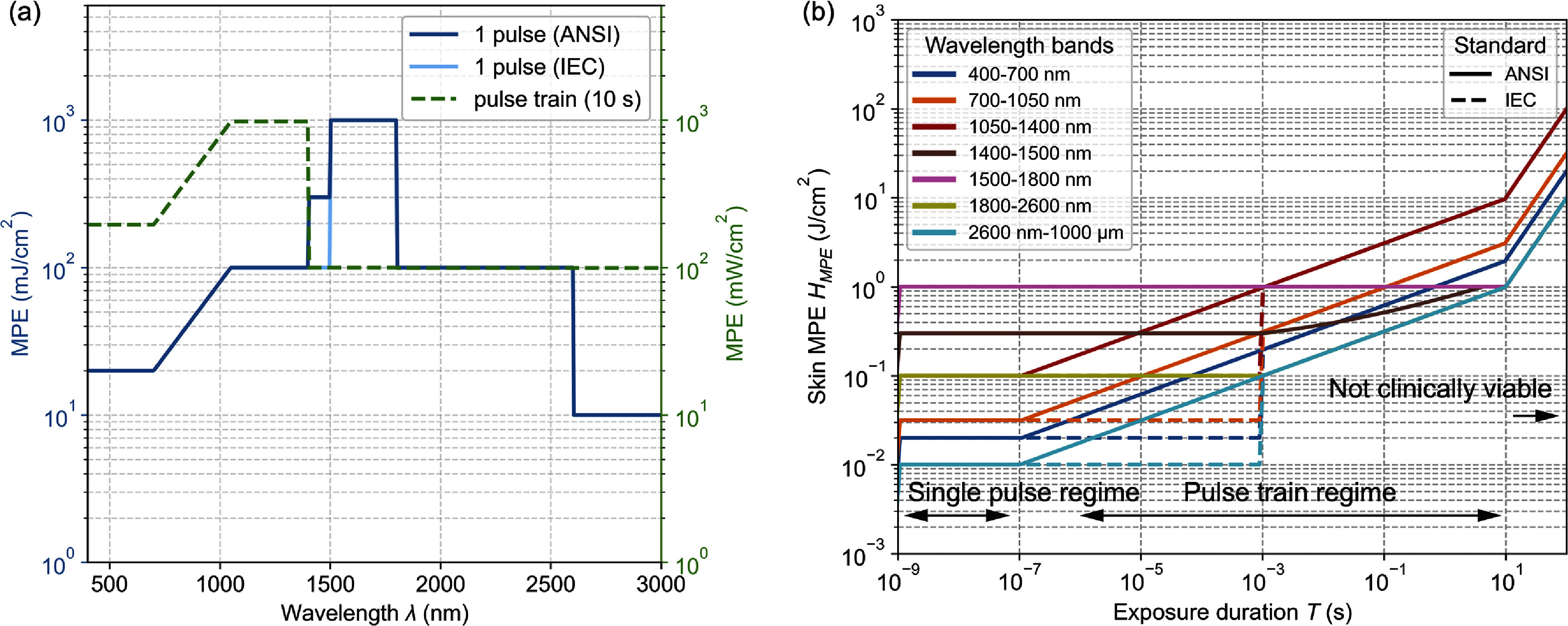
(a) Maximum Permissible Exposure (MPE) as a function of wavelength for single pulse exposure (blue, left axis) and for a 10 s pulse train (green, right axis), assuming nanosecond pulses. For the 10 s pulse train, the MPE is better expressed as an average irradiance. The IEC 60 825–1 2014 and ANSI Z136.1-2022 standards differ in the single pulse MPE in the 1400–1500 nm band. (b) MPE expressed as radiant exposure as a function of exposure duration for wavelength bands from 400 nm to 1000 $\mu$m. The repeated exposure regime (pulse train regime) is valid from exposure duration of $\mu$s and higher, as high-energy nanosecond-pulse light sources used in photoacoustics do not typically exceed repetition frequency of 100 s kHz. For exposure durations below a microsecond, only single pulses can typically be applied.

In practice, the deliverable per-pulse fluence $\Phi$, is limited by exposure standards that set a strict upper limit on the permissible fluence, which depends on the source wavelength but also on the pulse width $\tau_\mathrm{p}$, the exposure time $T$ and the laser PRF $f$ in the case of pulse trains [51–53]. In photoacoustics, except for ocular applications, skin exposure standards apply. International and American safety standards from the International Electrotechnical Commission (IEC) and American National Standards Institute (ANSI), as well as the International Commission on Non-Ionizing Radiation Protection (ICNIRP) exposure guidelines, provide MPE values for skin as a function of wavelength and exposure duration (figure 1).

In the case of repeated exposures, as is commonly the case in photoacoustics, the MPE is governed by two rules: the single-pulse limit (Rule 1) and the average irradiance (also referred to as average power) limit (Rule 2). The former prevents single pulse damage and the latter accounts for the cumulative thermal increase from repeated exposures. For Rule 1, the relevant exposure time is thus the pulse duration ($T = \tau_\mathrm{p}$), whereas for Rule 2, it is the duration $T$ of the pulse train. For both ANSI and IEC standards, MPE values are either given as radiant exposure $H$ (J cm$ ^{-2}$) for $10^{-9}\,\mathrm{s} < T < 10\,\mathrm{s}$ or laser irradiance $I$ (W cm$ ^{-2}$) for $ T > 10\,\mathrm{s}$. Laser irradiance can be converted to radiant exposure via $IT$, as was done in figure 1(b). For repeated pulse exposures ($N$ pulses in a train during a measurement window $T$), the MPE from Rule 2 can be converted to a per-pulse fluence limit:

\begin{align*} \Phi_{\mathrm{rule\,2}}\left(\lambda,f,T\right) = \begin{cases} \dfrac{H_{\mathrm{MPE}}\left(\lambda,T\right)}{fT}, &amp; 10^{-9} < T < 10~\mathrm{s}, \\[6pt] \dfrac{I_{\mathrm{MPE}}\left(\lambda\right)}{f}, &amp; T > 10~\mathrm{s}. \end{cases} \end{align*}

The effective fluence limit is the most restrictive of the two MPE values (Rules 1 and 2),

\begin{align*} \Phi_{\mathrm{eff}}\left(\lambda,\tau_\mathrm{p},f,T\right) = \min\left\{ H_{\mathrm{MPE}}\left(\lambda,\tau_\mathrm{p}\right), \; \Phi_{\mathrm{rule\,2}}\left(\lambda,f,T\right) \right\}.\end{align*}

We limit the following discussion to $T > 10^{-9}\,\mathrm{s}$ and $\lambda > 400\,\mathrm{nm}$, which are the most relevant ranges for biomedical photoacoustic imaging. Note that for ocular exposure in the visible and NIR spectral range, there is a third rule specifically for repetitively pulsed lasers. This rule takes into account PRF and the effective number of pulses being averaged. For retinal imaging, the most restrictive of the three rules applies. Photoacoustic ophthalmoscopy has been investigated [54]. For the purpose of this review, we will consider the exposure limits of skin only, as non-invasive imaging will usually irradiate the skin. Specific exposure limits for, e.g. mucosa or endothelium do not exist.

**Figure 2.**
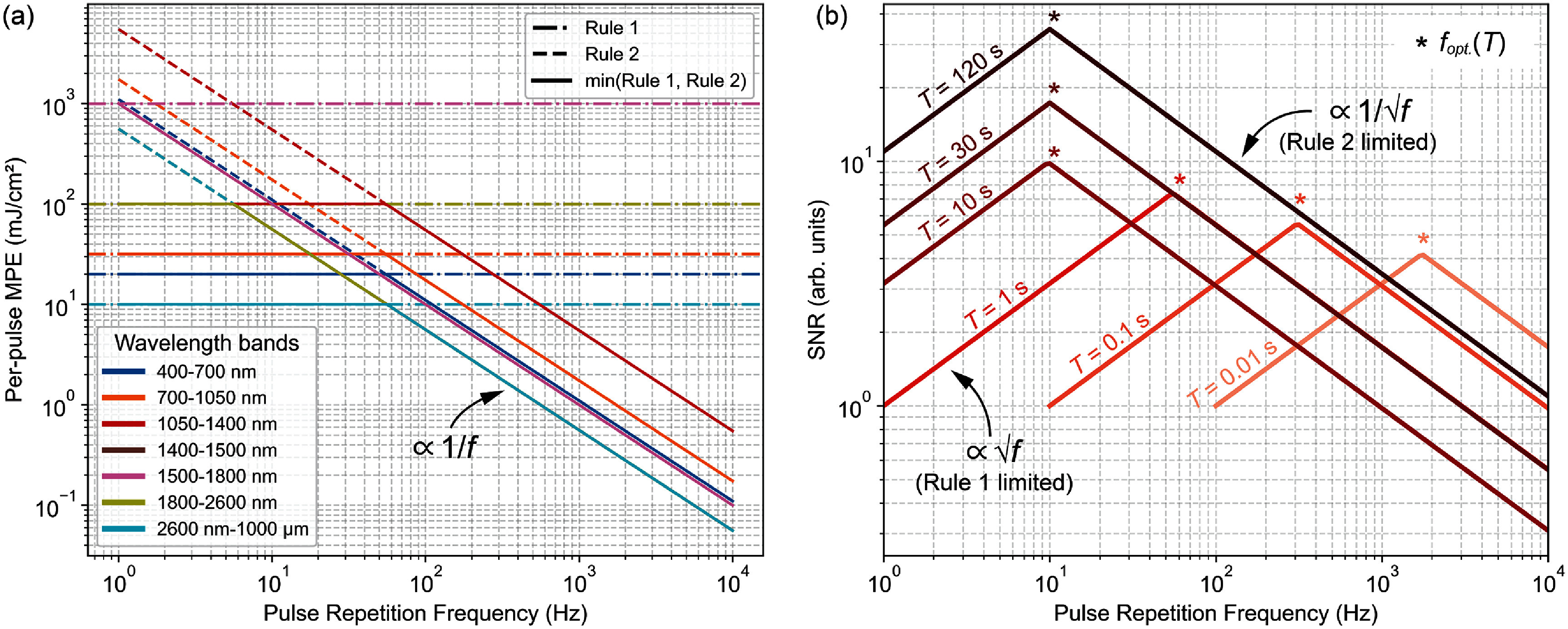
(a) Per-pulse MPE calculated for 10-ns pulses, a total exposure time of 1 s, as a function of pulse repetition frequency (PRF). Rule 1 dictates single-pulse MPE and is therefore independent of PRF. Rule 2 dictates total radiant exposure permissible during the exposure window, thus the per-pulse MPE is inversely proportional to PRF ($1/f$). MPE is the minimum of permissible exposure from Rules 1 and 2. (b) Safety-aware SNR as a function of total exposure time and PRF for an exemplary wavelength of 800 nm, highlighting the $\sqrt(f)$ dependence of the SNR at low PRF and 1/$\sqrt(f)$ at high PRF. The optimal PRF corresponding to the peak SNR at the regime transition, $f_\mathrm{opt}$, is indicated by a star. The ANSI standard is used to generate these plots [51].

For a given wavelength and a given integration time, the radiant exposure limit $H_{\mathrm{MPE}}$ is a constant. This implies that Rule 1 yields a constant MPE value, whereas Rule 2 gives a value that decreases as $1/f$, where the total allowed energy is evenly distributed between the pulses. This leads to a crossing point in $\Phi_{\mathrm{eff}}(f)$ between Rule 1-limited and Rule 2-limited regimes, illustrated in figure 2(a) for an exemplary exposure time of $1\,\mathrm{s}$ and a laser pulse duration of $10\,\mathrm{ns}$. Following $\Phi_{\mathrm{eff}}$ determination, we can then derive the safety-aware SNR by substituting (9) into (5), yielding:

\begin{align*} \mathrm{SNR}_N \propto \frac{\Gamma\,\mu_a\,\Phi_{\mathrm{eff}}\,\sqrt{N}}{\sigma_\mathrm{p}} = \frac{\Gamma\,\mu_a\,\Phi_{\mathrm{eff}}\,\sqrt{fT}}{\sigma_\mathrm{p}}.\end{align*}

For a given measurement window $T$, the SNR therefore follows a different scaling with PRF under each safety regime:

\begin{align*} \text{single-pulse limited (Rule 1):} \qquad &amp; \mathrm{SNR}_N \propto \sqrt{f},\end{align*}

\begin{align*} \text{average-power limited (Rule 2):} \qquad &amp; \mathrm{SNR}_N \propto \frac{1}{\sqrt{f}}.\end{align*}

This, in turn, suggests that an optimal SNR exists at the repetition frequency corresponding to the crossing point. In figure 2(b), the SNR is plotted for a single wavelength (800 nm) and several integration times $T$, as a function of PRF. The SNR is normalized to 1 for $N = 1$. Interestingly, assuming that sufficient energy budget is available to operate at the MPE and that a single measurement window is used for simplicity, integrating longer is not always beneficial. For instance, when using a 300 Hz laser, the optimal SNR is achieved with an optimal integration time of only $0.1\,\mathrm{s}$. This peak SNR cannot be recovered until integration times exceed $100\,\mathrm{s}$, in the regime where the MPE is defined as a constant average power ($ T > 10\,\mathrm{s}$), and at which point each pulse is limited to a much lower energy.

**Figure 3.**
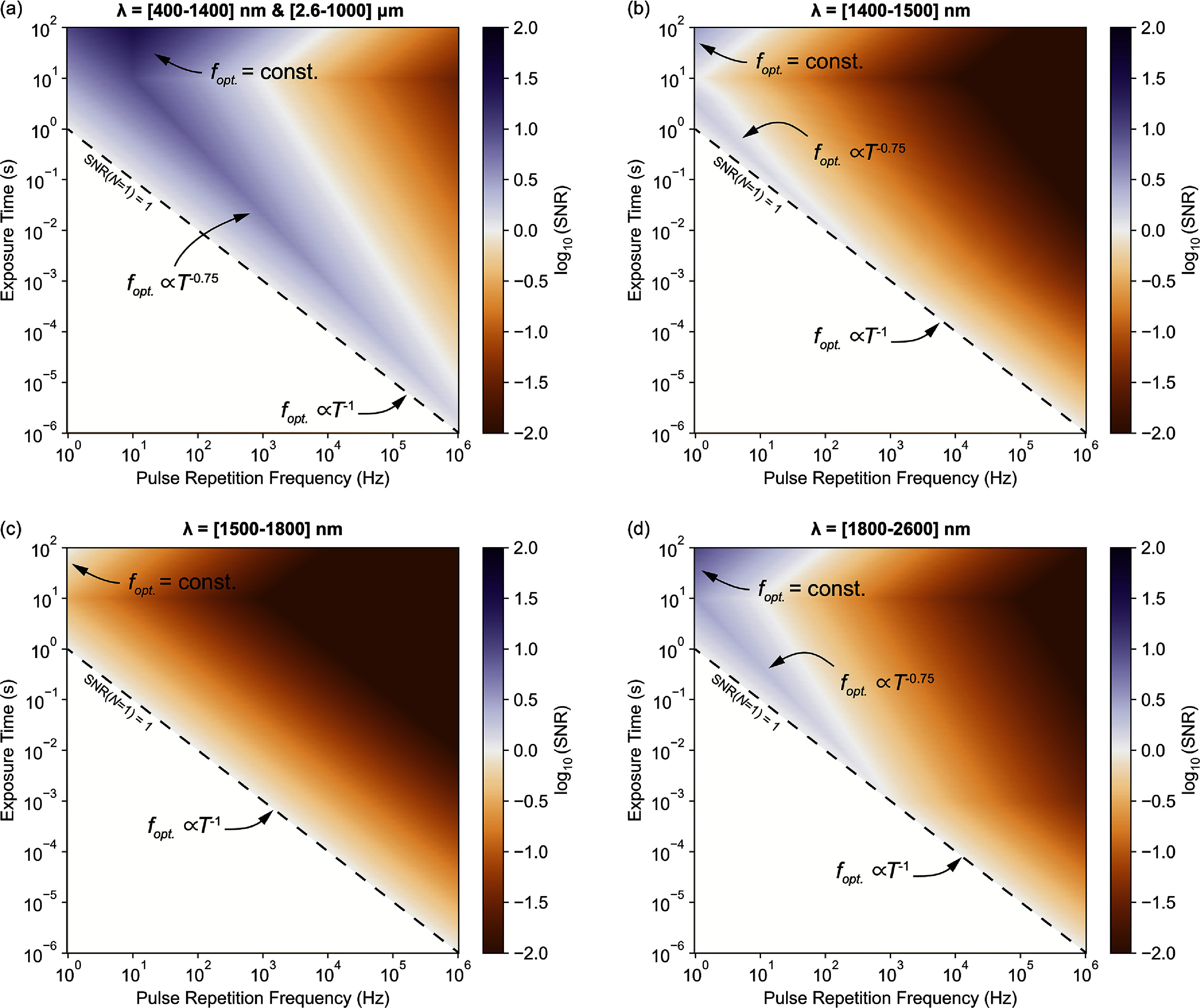
SNR maps as a function of PRF and exposure time and wavelength bands (a)–(d), relative to a References SNR taken as 1 for a single pulse exposure ($N$ = 1). Except for the 1500–1800 nm band (c), there exists a locus ($f,T$) defining operation points maximizing SNR. The ANSI standard is used to generate these maps.

Beyond the single wavelength example, we can generalize this analysis across the different wavelength bands defined by the IEC and ANSI standards. In figure 3, each panel shows the SNR as a function of PRF and exposure time, assuming operation at the MPE limit, under the ANSI standard. Analysis using the IEC standard leads to similar results with subtleties that do not affect the general interpretation of these plots. For all panels and all wavelengths, the SNR was normalized such that SNR = 1 when $N$ = 1. This normalization removes the absolute scaling of the MPE across wavelength regions but conserves the relative scaling of the MPE values across exposure times. As a result, the SNR($f$, $T$) maps can be similar for bands that are treated separately in the exposure standards (e.g. 400–1400 nm and 2.6–1000 $\mu$m, figure 3(a)). Except for the 1500–1800 nm band, there exists a locus ($f$, $T$) that maximizes SNR ($\mathrm{SNR} > 1$), forming a ridge on the SNR maps. This ridge follows the boundary separating the single-pulse-limited regime (Rule 1) from the average-power-limited region (Rule 2) as explained earlier. In the bands where the ridge appears for $T < 10$ s, figures 3(a)–(c), the average power limit increases non-linearly with time. The optimal repetition frequencies scale as $T^{-0.75}$ departing from SNR = 1 and giving the opportunity for SNR maximization. For $T > 10$ s, the MPE transitions to a constant average-power limit, and the ridge remains at a constant repetition frequency. From a practical standpoint, these maps indicate that once the imaging wavelength(s) have been determined, the PRF and exposure time can be jointly optimized. For example, for a system in which the laser operates at a fixed repetition frequency, one can maximize the image SNR by selecting the exposure time that suffers least from safety constraints to best benefit from averaging. If the exposure time is the main constraint and the laser allows a tunable repetition frequency, it can be adjusted to reach the maximum SNR corresponding to that exposure time limit. Generally, the analysis provides a direct approach for selecting operation points that best compromise repetition frequency and exposure time to optimize SNR while respecting the ANSI/IEC limits.

#### 1.2.4 Repetition frequency and frame rate

For raster scanning systems, requiring $N_{\mathrm{pos}}$ A-lines per frame, $N_\lambda$ wavelengths, and $N_{\mathrm{avg}}$ averages per A-line, the achievable frame rate is approximately:

\begin{align*} R_{\mathrm{frame}} \approx \frac{f}{N_{\mathrm{pos}}\,N_\lambda\,N_{\mathrm{avg}}}.\end{align*}

Under such exposure conditions, the MPE considerations must be reviewed carefully as the tissue may be exposed to non-uniformly spaced pulse trains where not only the laser repetition frequency must be considered but also the revisit period of the laser beam at the same tissue location.

In array-based PACT where one pulse irradiates a large volume to form a 2D frame, the $R_{\mathrm{frame}} \unicode{x2A7D} f/(N_\lambda$ $N_{\mathrm{avg}}$), is still limited by $f$ and any averaging budget [5].

## 2 Light source technologies for PA imaging

This section surveys the major families of pulsed light sources that have been applied for PA imaging. Table 1 provides a very succinct summary of the same information.

**Table 1.** Comparison of light source technologies for photoacoustic imaging (typical values).

Technology	Wavelength (nm)	Pulse dur. (ns)	Pulse energy	Max rep. rate	Beam quality	Size	Wall-plug eff. (%)	Cooling	Cost^a^	References
Q-switched Nd:YAG										
High power	532, 1064	2–10	100–250 mJ	10–200 Hz	Top hat	Benchtop/cart	$\sim$0.5–3	Water-to-air	$\unicode{x20AC}\unicode{x20AC}\unicode{x20AC}\unicode{x20AC}$	[118, 119]
High PRF	532, 1064	2–10	10–500 $\mu$J	1–50 kHz	Near TEM$ _{00}$	Benchtop/Shoebox	$\sim$5–15	Air	$\unicode{x20AC}\unicode{x20AC}\unicode{x20AC}\unicode{x20AC}$	[57, 59]
Nd:YAG+OPO	$\sim$410–2600 (tunable)	3–8	Up to 20% of pump, varies with $\lambda$	same as pump	Multimode	Benchtop/cart	$\sim$0.1–2	Air/Water-to-air	$\unicode{x20AC}\unicode{x20AC}\unicode{x20AC}\unicode{x20AC}\unicode{x20AC}$	[63–66]
Laser diodes	750–1064 (discrete)	10–200	0.01–7 mJ	1–50 kHz	Multimode	Handheld/Shoebox	$\sim$20–40	TEC^b^	$\unicode{x20AC}\unicode{x20AC}\unicode{x20AC}\unicode{x20AC}$	[78, 80, 85–87]
LED arrays	405–980 (discrete)	30–150	1–200 $\mu$J	1–16 kHz	Diffuse	Handheld	$\sim$20–30	Passive	$\unicode{x20AC}\unicode{x20AC}\unicode{x20AC}$	[11, 90, 91, 96]
VCSEL arrays	760–940 (discrete)	100–300	1–10 $\mu$J	1–3 kHz	Near-Gaussian	Handheld	$\sim$30–55	TEC	$\unicode{x20AC}\unicode{x20AC}$	[46, 99]
Fibre MOPA (Yb / Er / Tm)	1030–1100, 1520–1600, 1650–2050^c^	1–12	0.1–10 $\mu$J	100 Hz-MHz	Single-mode	Shoebox	$\sim$10–20	Air/TEC	$\unicode{x20AC}\unicode{x20AC}\unicode{x20AC}\unicode{x20AC}$	[102, 103, 105, 106, 108]
Supercontinuum sources	500–2400 (filtered)^d^	3–7	0.1–30 $\mu$J^e^	MHz	Single-mode	Benchtop	$\sim$5–20	Air/TEC	$\unicode{x20AC}\unicode{x20AC}\unicode{x20AC}\unicode{x20AC}$	[74, 109, 110]
Raman / SRS-shifted sources	Discrete and switchable^f^	1–10	0.1 $\mu$J–2 mJ	Same as pump	Single-mode	Benchtop	$\sim$20–32	Air/TEC	$\unicode{x20AC}\unicode{x20AC}\unicode{x20AC}\unicode{x20AC}$	[67–69, 112–115]

^a^ Number of $\unicode{x20AC}$ signs is the approximate $ ^{10}$log of the system acquisition cost.

^b^ Thermoelectric cooling.

^c^ Some systems offer a degree of tunability within these bands.

^d^ Broadband source and spectrally filtered for wavelength selection.

^e^ The energy available in a selected spectral band is much lower than the total output pulse energy. $ < 1\%$ efficiency for wavelength selection.

^f^ Discrete Raman-shifted wavelengths depend on the pump source, Raman medium, and Raman order.

### 2.1 Q-switched solid-state lasers and Nd:YAG-pumped OPOs:

Q-switched Nd-doped Yttrium Aluminium Garnet (Nd:YAG) lasers are the classical workhorse sources for PA imaging. In these lasers, a neodymium-doped YAG crystal in a lossy cavity is pumped by a flashlamp or diodes to build inversion at 1064 nm, where the large cavity loss allows for a very high gain to accumulate. A fast-switching optical modulator, usually a Pockels cell or an acousto-optical modulator, rapidly flips the cavity $Q$-factor to a high value, upon which the excess gain is extracted in one high-energy pulse with a duration equal to the cavity round-trip time (typically 2–10 ns) and narrow linewidth (typically $ <$0.5 nm). Nonlinear stages provide fixed harmonics at 532, 355 and 266 nm. Early tomography and microscopy systems used such sources [37, 40]. Flashlamp-pumped Nd:YAG systems deliver tens to several hundreds of mJ per pulse at 1064 nm at 10–50 Hz, with lower energy at the harmonics and a reasonable beam quality with, e.g. a top-hat profile. These parameters favour single-pulse-limited operation for deep illumination of centimetre-scale fields at fluences close to the skin MPE. The wall-plug efficiency is low, around 1% [55, 56]. These lasers require water cooling and have a large footprint for both the laser head and power supply.

Diode-pumped solid-state (DPSS) Nd:YAG lasers trade pulse energy for higher PRF and compactness. Typical systems provide 1064 nm, 3–8 ns pulses, good beam quality ($M^2 \approx 1$–2) and sub-nanometre linewidth. Pulse energy and PRF are coupled, with typical figures of 100 s of mJ pulse energy at 100–200 Hz PRF, to energies of 10–100 s of $\mu$J at PRFs in the kHz–10 s of kHz range. Electrical-to-optical efficiency can reach about 10%–15%, with water or forced-air cooling and much smaller heads suitable for clinical carts [57, 58].

Optical and acoustic resolution microscopy, and endoscopy PA systems can further benefit from the higher pulse repetition frequencies offered by DPSS lasers [59–61]. As these technologies accumulate their image by point scanning, sampling a volume requires many acquisitions. High laser PRFs of several to tens of kHz improve acquisition speed and expand the possibilities for averaging. An upper limit to the useful PRF is the acoustic delay time at the desired imaging depth (see equation (7)). For OR-PAM, which has a very short viewing depth, PRFs exceeding 1 MHz have been used for scanning cm$ ^2$ areas at 6 $\mu$m resolution in a few seconds [62].

OPOs extend these fixed wavelengths to a continuum of wavelengths. A nonlinear crystal in a resonant cavity, pumped by 1064, 532, or 355 nm Nd:YAG pulses, splits each pump photon into signal and idler photons. Each of the signal and idler photons has a longer wavelength than the pump, and their energies add up to the pump photon energy. By changing the phase-matching condition, for example by crystal rotation or temperature tuning, the signal can be tuned typically from about 660–1300 nm for a 532 nm pumped system (extended throughout the visible down to $\sim$410 nm when pumping with 355 nm), while the idler can reach $\sim$2.6 $\mu$m [63]. Commercial nanosecond OPOs for PA imaging provide roughly 20–180 mJ per pulse in the NIR at 10–200 Hz with 3–8 ns pulses, linewidths of order 1–5 nm, highly multimode beams and about 20%–40% optical conversion efficiency. The pump laser, OPO head, chiller, control electronics and fibre delivery can be integrated in a cart-sized system [64]. Modern DPSS-pumped OPOs also support fast electronic wavelength switching, including shot-to-shot selection of discrete wavelengths [64]. This pulse-to-pulse tunability allows each consecutive pulse to probe a different wavelength without reducing frame rate, which is advantageous for multispectral PA imaging and spectral unmixing, as shown in multispectral PAM and handheld MSOT [65]. Specialized high-repetition-rate OPO designs trade pulse energy for speed, reaching $\sim$kHz PRF with microjoule- to millijoule-level pulses, which can be attractive when average-power MPE, rather than single-pulse fluence, limits exposure, for example in high-speed OR-PAM and intravascular PA imaging [66].

While OPOs offer the greatest wavelength versatility, other methods for wavelength conversion from ‘standard’ wavelengths, available from Nd$ ^{3+}$-doped crystal lasers, have been explored. Sacrificing tunability, the resulting laser systems are less complex than OPOs, offer a higher conversion efficiency, and are potentially cheaper. Stimulated Raman scattering (SRS) shifts the pump wavelength by a fixed energy to (usually) longer wavelengths. Many different Raman-active crystals exist, such as Ba(NO$ _3$)$ _2$, which has been used for lipid imaging at 1197 nm, shifted by 1047 cm$ ^{-1}$ from 1064 nm [67, 68]. SRS in optical fibres provides a 440 cm$ ^{-1}$ shift in photon energy, and amplifying the second Stokes line at 880 cm$ ^{-1}$ allowed quantification of blood oxygenation by combining the pump wavelength of 532 nm and the SRS wavelength of 558 nm in OR-PAM [62]. As many as five distinct wavelengths could be generated by Raman-shifting a 532 nm pulse in silica fiber, separated by optical delay of 100 s of ns, enabling imaging of arteries and veins, lymphatics with the aid of a dye, and blood flow velocity by combining multiple pulses at isosbestic points in the Hb/HbO$ _2$ absorption spectra [69]. Pumping a dye or Ti$ ^{3+}$:Al$ _2$O$ _3$ laser by the green output of a Nd:YAG pulsed laser provides a limited range of wavelength tunability [59, 70, 71].

Other Nd$ ^{3+}$-doped crystals, such as Nd:YLF or Nd:YVO$ _4$ may be suitable, usually resulting in slightly different output wavelengths. Nd:YLF is more efficient than Nd:YAG at kHz PRF applications, and has been used for pumping a dye laser, SRS amplifier, or supercontinuum (SC) source in OR-PAM setups [70, 72–74].

One other solid-state laser that is worth mentioning is the Alexandrite (Cr$ ^{3+}$:BeAl$ _2$O$ _4$) laser, operated by flashlamp pumping or in a DPSS configuration. Alexandrite lasers are commonly used for hair removal. With pulse energies of typically 100 mJ, Alexandrite lasers are wavelength tunable between 700 and $&gt;$800 nm, with maximum output at 755 nm [75]. It has been combined with the fundamental wavelength from an Nd:YAG laser for clinical breast imaging [76].

### 2.2 LDs and LD arrays or stacks

LDs are electrically driven semiconductor lasers, most often InGaAs/AlGaAs emitters in the 750–980 nm range [77–79], with emerging devices $&gt;$1000 nm [80–82]. They are driven with $\sim$10–200 ns current pulses at 1–50 kHz PRF as single emitters, 1D bars or 2D stacks. Gain-switched diodes can be pulsed at arbitrary PRFs, fully controlled by the current pulses from the driver. Overdriven CW single-emitter LDs driven by a pulsed current source can produce pulses with an energy up to 200 nJ and a pulse width $ <$10 ns [83]. Several wavelengths across the visible and NIR spectrum were demonstrated. LD bars were employed in a microscopy setup, providing 16 $\mu$J, $ < $ 50 ns pulses at 905 nm. Careful beam conditioning of the extended source achieved a lateral resolution of 21 $\mu$m [84].

Tomographic PA systems can produce good quality images with pulse energies of $\sim$1–7 mJ per pulse, averaging pulses at the kHz repetition rate of LDs [8, 78, 83, 85, 86]. As the achievable peak power in semiconductor gain media is limited, sufficient pulse energy is typically obtained only by extending the optical pulse duration to values on the order of, or exceeding, 100 ns [87]. Such pulse durations are significantly longer than the stress relaxation time of small absorbers in soft tissue. This substantially reduces the efficiency of photoacoustic signal generation and suppresses the high-frequency components required to resolve small features, making objects below this size difficult to detect reliably. Longer pulse might be useful in transcranial imaging, where lower-frequency acoustic components reduce attenuation from the skull with a trade-off in small-feature contrast [87]. The spectral linewidth is usually a few nanometres, which is spectrally narrower than most PA chromophores. Beam quality is the main optical compromise: LD emitters and stacks produce highly divergent, astigmatic, strongly multimode beams (large $M^2$) that require optics for collimation and homogenization, and are usually coupled into short-focal-length lenses or multimode fibres with high NA [7]. In contrast to Nd:YAG/OPO systems, LDs offer high electrical-to-optical conversion efficiency (typically 20%–30%), compact cm-scale heads, and mostly thermoelectric cooler (TEC) or forced-air cooling, so complete PA light modules can be handheld or shoebox-sized and are typically an order of magnitude cheaper than class-IV solid-state lasers [86, 88]. As these devices are often operated with overdriven current pulses at high repetition rates, thermal management is critical for maintaining output stability and operational lifetime [89]. LD-based PA systems typically trade single-pulse fluence and penetration depth for very high PRF and low cost, relying on frame averaging to recover SNR [80].

### 2.3 LEDs

**Figure 4.**
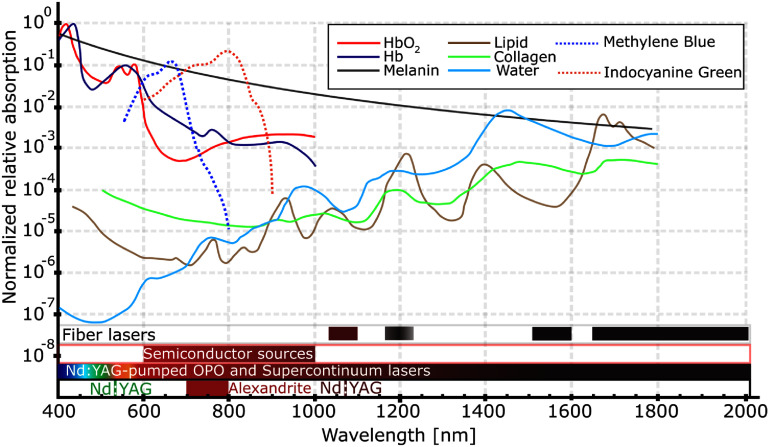
Normalized optical absorption spectra of key chromophores and the availability of light sources.

High-power pulsed LEDs are electrically driven, incoherent, broadband nanosecond sources, conceptually similar to LDs but with spontaneous rather than stimulated emission. Commercial PA systems typically use NIR LEDs in the 620–980 nm range (often 750 and 850 nm), with some implementations at 405–530 nm for superficial imaging and spectroscopy [7, 11, 43, 90]. The spectral linewidth is broad, typically 20–40 nm FWHM, which is still acceptable for most PA applications because endogenous chromophore spectra change slowly over the NIR wavelength range [7, 43] (see figure 4).

Modern LED arrays are heavily overdriven with 30–150 ns current pulses at 1–16 kHz repetition frequencies [11, 89–92]. Longer pulse width has shown to achieve more pulse energy, enhancing the imaging depth at the cost of resolution [93]. A typical near-infrared LED array operating around 750–850 nm delivers pulse energies on the order of 50–200 $\mu$J per array. For example, an 850 nm LED array with an emitting area of $5 \times 1$ cm can deliver up to 200 $\mu$J per pulse with a pulse duration of approximately 70 ns, at repetition rates in the range of 4 kHz [11, 90–92]. These parameters support photoacoustic frame rates of a few tens of Hz, depending on the degree of signal averaging. Wavelength tunability per LED is limited to small thermal or current shifts, so multispectral imaging is achieved by combining two or more discrete LED wavelengths (for example, 750/850 nm or 820/940 nm) and firing them sequentially [11, 91, 94]. Beam quality is diffuse and nearly Lambertian, with large divergence angles ($\sim\!\!120^\circ$), usually delivered without fibre delivery [90, 95]. Electrically, LEDs are efficient with electrical-to-optical efficiency of a few tens of percent and can be mounted on compact cm-scale boards with passive heatsinks or small fans. LED-based PA systems have generally shown stable operation over extended use [11, 96]; however, adequate thermal management remains important for maintaining output stability and device lifetime [97]. Complete LED-based PA heads, including two to four arrays and a linear US probe, are handheld or probe-sized and are cheap and eye-safe, as they deliver diffused, low-energy light [7, 11, 95]. The trade-off is lower per-pulse energy than LDs and orders of magnitude less than Nd:YAG/OPO systems, which limits imaging depth, thus high PRF and sensitive detection are needed for deep tissue imaging. In practice, LED sources are best suited for shallow vascular and functional imaging ($\unicode{x2A7D}$2 cm in soft tissue), high-frame-rate monitoring and low-cost or point-of-care systems where portability, safety and price outweigh imaging depth and broad wavelength coverage.

### 2.4 Vertical-cavity surface-emitting lasers (VCSELs)

VCSELs are compact semiconductor lasers in which a short cavity between distributed Bragg reflectors emits a circular beam normal to the wafer surface [98]. GaAs-based VCSEL arrays for PA imaging typically operate in the 760–940 nm range, most often near 850 nm where haemoglobin absorption is high, and tissue penetration is favourable [46, 98, 99]. VCSELs are typically driven by nanosecond electronic current pulses at kHz repetition frequencies, with peak powers of several tens of watts per emitter and pulse energies of a few microjoules (for example $\sim$40 W, 200 ns, 3 kHz, $\sim$8 $\mu$J per pulse in a wearable PA patch) [46, 99]. The optical linewidth is narrow, typically well below 1 nm FWHM in single-mode devices and only a few nanometres in high-power arrays, giving much higher wavelength spectral selectivity [98]. Beams are close to Gaussian with low $M^2$ and symmetric divergence of about 10$ ^\circ$–20$ ^\circ$, which simplifies collimation, fibre coupling, and uniform illumination over centimetre-scale fields of view [98]. Electrical-to-optical power-conversion efficiencies of about 30%–45% are typical for 850–940 nm VCSEL arrays, while recent multi-junction devices have demonstrated pulsed power-conversion efficiencies above 70% [100]. These characteristics have enabled compact handheld PA probes and high-power VCSEL subsystems for perfusion and oxygenation imaging, as well as soft wearable patches that co-integrate VCSEL and ultrasound arrays, achieving centimetre-scale penetration and Hz-class frame rates after averaging while remaining within thermal and safety limits at kilohertz PRFs [46, 99, 101]. A current drawback is the limited set of commercially available high-power VCSEL wavelengths, which restricts the multiwavelength use for PA imaging.

### 2.5 Fibre-based light sources

Fibre-based light sources leverage rare-earth—doped silica fibre as the gain medium and provide compact, alignment-free architectures with excellent beam quality (typically $M^2 < 1.2$), flexible pulse formats, and high electrical-to-optical efficiency. The accessible wavelengths are determined by the dopant species of the gain fibre. When combined with additional amplification stages in a master-oscillator power-amplifier (MOPA) configuration, these fibre sources can deliver pulse energies sufficient for photoacoustic excitation. Yb-doped fibre lasers are widely used as high-power pumps or nanosecond excitation sources due to their high gain, low quantum defect, and watt-to-kilowatt scalability in the 1.03–1.10 $\mu$m region. Typical nanosecond Yb-fibre lasers provide tens of microjoules with 1–10 ns pulses at hundreds of kilohertz repetition frequencies and have been applied to photoacoustic imaging of melanoma [102]. Similarly, Er-doped fibre lasers can deliver $\mu$J-level nanosecond pulses at kHz repetition frequencies covering the 1.52–1.60 $\mu$m region. A Q-switched Er-doped fibre laser operating at 1600 nm produced $\sim$12 ns pulses with up to 2.4 $\mu$J at 100 kHz and successfully generated photoacoustic signals from *ex vivo* tissue [103]. Tm-doped fibre lasers extend coverage into the 1.65–2.05 $\mu$m region and have demonstrated lipid-selective PAM operation in the 1.7 $\mu$m wavelength band, delivering nanometre-level linewidths and spectral energy densities up to hundreds of nJ nm$ ^{-1}$ [104, 105]. These fibre lasers, when combined with harmonic generation units, can be operated from the ultraviolet to the near-infrared depending on the fundamental wavelength [106, 107]. Commercial nanosecond fibre MOPA systems reach electrical-to-optical efficiencies around 20% in compact air- or TEC-cooled packages [108].

In wavelength regions where rare-earth gain media are unavailable, two principal nonlinear fibre-based approaches are widely used to generate photoacoustic excitation wavelengths: SC generation and SRS. SC sources rely on nonlinear broadening mechanisms, self-phase modulation, four-wave mixing, soliton fission, and Raman interactions when picosecond or nanosecond pulses are launched into highly nonlinear or photonic-crystal fibres. The resulting broadband spectra, spanning hundreds of nanometres, are spectrally selected using bandpass filters such as linear-variable filters or acousto-optic tunable filters, yielding wavelength-tunable nanosecond sources. For lipid-selective PAM in the 1650–1850 nm band, 1.55 $\mu$m-pumped SC lasers using 3–7 ns pulses at 30–100 kHz deliver $\sim$18–30 $\mu$J per pulse into the fibre and generate 1440–1950 nm SC with spectral energy densities of $\sim$25 nJ nm$ ^{-1}$ at $\sim$1.7 $\mu$m; after filtering into 20–40 nm bands, this yields hundreds of nJ per pulse at each wavelength for high-resolution spectroscopic PA and multispectral PAM of lipids *ex vivo* and *in vivo* [109, 110]. Although SC sources offer exceptional tuning flexibility at kHz-class PRFs with diffraction-limited beam quality, their broad spectral spread results in modest spectral energy density (typically a few tens of nJ/nm) at a given wavelength.

In contrast, SRS-based wavelength conversion provides much narrower linewidths and significantly higher spectral energy density. When driven by high-energy nanosecond pump pulses, Raman-gain media (typically undoped silica fibre) generate discrete Stokes wavelengths with typical linewidths of $&gt;$1 nm [111]. Recent studies have shown that injecting an additional narrow-linewidth Raman seed ($<$0.1 nm) into the SRS process maintains sub-0.1 nm spectral purity throughout the cascade, greatly improving stability and spectral density, an approach demonstrated for high-purity 1.2 $\mu$m and 1.7 $\mu$m photoacoustic excitation [112, 113]. Compared with SC systems, SRS sources sacrifice broadband tunability but offer superior spectral purity and higher energy per spectral band, making them particularly attractive for high-contrast, lipid-specific PAM in these wavelength regions, where narrow linewidth and high pulse energy directly influence sensitivity and quantitative performance [113]. In intravascular PA imaging, a 1064 nm MOPA was used to pump a Ba(NO$ _3$)$ _2$ Raman crystal, generating 1197 nm pulses of 3.7 ns duration with 2.0 mJ energy and 32% conversion efficiency for lipid-specific IVPA [68].

Multispectral PAM images can be achieved through the wavelength switchability of an SRS amplifier, with switching rates of hundreds of kilohertz in the NIR spectral range [114, 115]. In addition, dual-wavelength hybrid light source architectures that combine 532 nm excitation with fibre-based SRS regimes have been employed to quantify blood SO$ _2$ levels or exogenous contrast (e.g. nanoparticles) through multispectral photoacoustic measurements [116, 117].

## 3 Light source selection for different applications

### 3.1 Wavelength selection and chromophore contrast

Optimal wavelength selection is central to maximizing contrast and quantitative accuracy in photoacoustic imaging. Excitation wavelengths are typically chosen close to absorption maxima or steep spectral slopes of the target chromophores. Table 2 summarizes the absorption features and commonly used wavelengths for the main endogenous and exogenous chromophores. Figure 4 summarizes the spectral absorption of the main endogenous and clinically relevant exogenous chromophores used for *in vivo* photoacoustic imaging and overlays these with the wavelength coverage of commonly available light sources.

**Table 2.** Chromophores or contrast agents for clinical or translational photoacoustic imaging, reported excitation ranges and suggested optimal wavelengths.

Chromophores	Photoacoustic targets	Optimal / widely used wavelength (nm)	References
Endogenous chromophores			

Oxygen saturation (sO$ _2$)	Blood oxygenation, hypoxia, perfusion	700–900 nm (e.g. 750/850, 730/850, 750/800/850)	[132–134]
Haemoglobin (total, HbT)	Vascular morphology, blood volume	750, 800, 850, 1064	[132–134]
Melanin	Melanoma, pigmented lesions, skin mapping	532, 680, 730, 800	[135, 136]
Lipids	Atherosclerotic plaque, adipose / fat, myelin	930, 1210, 1720	[13, 137, 138]
Water	Tissue hydration, edema, background absorber	970, 1200, 1450	[13, 14]
Collagen	Fibrosis, connective tissue, breast stroma, tendon	1200, 1550, 1700	[126, 139]

Approved exogenous contrast agents			

Indocyanine green (ICG)	Lymphatics, sentinel nodes, perfusion, liver	780, 800, 805, 860	[128, 140]
Methylene blue (MB)	Sentinel nodes, lymphatics, tumor marking	650, 665, 700, 710	[131, 141]

Haemoglobin is the dominant absorber in the visible and NIR and remains the primary endogenous target for clinical photoacoustic imaging. Oxy- and deoxyhaemoglobin exhibit strong absorption in the 500–600 nm range and distinct spectral shapes between 700 and 900 nm, with multiple isosbestic points including one near 800 nm where total haemoglobin can be estimated independent of oxygenation [12, 120]. Practical oximetry implementations typically use one wavelength on each side of this isosbestic point within the 700–900 nm window to maximize the difference in molar extinction coefficients while maintaining penetration depth and allowable fluence [12, 121]. In contrast, extending from dual- to multi-wavelength acquisitions further improves $\mathrm{sO_2}$ quantification and reduces bias from spectral colouring and unknown tissue composition [122, 123].

Melanin shows broadband, monotonically decreasing absorption from the visible into the NIR and is exploited for dermatology, ocular, and melanoma imaging, typically using visible or NIR wavelengths where melanin contrast is high at the expense of penetration depth [121, 124], but aided by the fact that melanin is typically superficial to tissues. Lipids and water have relatively featureless spectra in the conventional 700–900 nm NIR window, but exhibit strong, well-separated overtone bands between 970 and 2100 nm. Lipid absorption peaks around 1210 and 1700–1720 nm and water peaks near 1200, 1450, and around 1900 nm [120]. Excitation at wavelengths above 1000 nm at a small set of wavelengths enables tri-wavelength concentration mapping that quantitatively separates lipid and water several millimetres deep [125]. Collagen exhibits photoacoustic absorption features overlapping those of water and lipids, with effective collagen-dominated peaks around 1200, 1550, and 1700 nm in the longer wavelength NIR window [126]. Machine-learning-based unmixing of multi-wavelength imaging has been shown to improve collagen specificity in fibrotic and tumour stroma compared with linear spectral unmixing [127].

Clinically approved exogenous dyes follow the same principles. Indocyanine green (ICG) has a broad NIR absorption band whose peak shifts from about 780 nm in aqueous solution to around 800–805 nm when bound to plasma proteins; photoacoustic ICG imaging therefore uses wavelengths within roughly 740 to 900 nm to combine strong absorption with favourable penetration depth [128, 129]. Methylene blue (MB) absorbs strongly in the red to NIR region with a maximum near 660–670 nm, and photoacoustic implementations typically excite within 650–710 nm [130, 131]. Dual-wavelength methods using one wavelength near the MB peak and a second wavelength in the NIR window, for example, 710 and 870 nm, enable quantitative separation of MB from the haemoglobin background in phantoms and *ex vivo* tissues [130].

Across endogenous and exogenous targets, wavelength sets are best designed together with the spectral unmixing strategy. Fast dual-wavelength methods provide real-time contrast enhancement for guidance tasks, such as dye-based lymphatic mapping or margin assessment, at the cost of quantitative accuracy [128, 130]. Multi-wavelength approaches that sample 5–10 chosen wavelengths across the relevant spectra, combined with eigenspectra or machine-learning-based unmixing, have demonstrated improved robustness to spectral colouring and model errors in quantitative $\mathrm{sO_2}$ and collagen estimation *in vivo* [123, 127]

Wavelength selection must respect practical constraints. Scattering is lower for longer wavelengths in the NIR window and can increase penetration, but light also encounters stronger water absorption in that spectral range and more restrictive MPEs in parts of the spectrum [120, 121]. For haemoglobin and the clinically approved dyes in the NIR window, the 700–900 nm window remains a good compromise between contrast and depth, whereas lipids, water, and collagen benefit from targeted excitation in the 1100– 1900 nm range [120].

### 3.2 Light source requirements for different imaging regimes

#### 3.2.1 Photoacoustic tomography (2D and 3D)

In photoacoustic tomography, each laser pulse should illuminate a large tissue volume to form a 2D frame (or a 3D volume with suitable detection geometry). The light source, therefore, needs to deliver near-uniform fluence over a centimetre-scale field of view while still providing enough per-pulse energy to reach the targeted depth. This typically favours nanosecond sources in the mJ to 100 mJ class, where deep illumination can be achieved without relying excessively on frame averaging [3, 47, 49, 64].

Some of the key requirements are: First, tomography benefits from wide-field, homogenized illumination to reduce fluence hot spots (safety) and spatial bias (quantification). Practical implementations often use fibre delivery for high-energy lasers, but large fibre bundles can be bulky and reduce probe manoeuvrability [142, 143]. Second, imaging depth requirements push pulse energy upward because local fluence decays rapidly with depth. Q-switched Nd:YAG and Nd:YAG-pumped OPO systems commonly provide tens to hundreds of mJ per pulse (often at 10–100 Hz), making them suitable for deep, large-FOV tomography and multispectral studies when wavelength access is needed [3, 64]. Third, the PRF must avoid overlap of acoustic time-of-flight windows, giving an upper bound ($f \unicode{x2A7D} c_\mathrm{s}/h$) (for example, $\approx 150$ kHz at 1 cm depth). Note that this upper bound does not consider the time required for echo imaging that is interleaved with PA acquisition. In practice, PRF is usually much lower because high pulse energy sources run at tens to hundreds of Hz, and because average-power MPE limits become too restrictive as PRF increases.

For array-based PACT, frame rate is directly limited by PRF (and by the number of wavelengths and averaging) [5, 49–53]. For tomography using handheld probes, as is more likely to be translatable to a clinical setting, compact illumination is often the bottleneck. Diode lasers can be integrated into probes and can potentially be fibre-delivered with suitable coupling optics, whereas LEDs are without fibre delivery and can be mounted directly on the probe [10, 86, 95, 144]. High-power pulsed LED arrays (typically 1–16 kHz) deliver tens to hundreds of $\mu$J per pulse per array and are best suited to shallow tomographic imaging (about ${\unicode{x2A7D}} 2$ cm in soft tissue), where portability, cost, and simplified safety can outweigh maximum depth [11, 90, 91].

#### 3.2.2 PAM

PAM achieves microscopic resolution by confining optical excitation and acoustic detection to a small focal volume [12]. Depending on whether the lateral resolution is determined by optical focusing (optical-resolution PAM; OR-PAM) or by the acoustic detector (acoustic-resolution PAM; AR-PAM), the optical beam requirements, and therefore the light source specifications, differ substantially from those of photoacoustic tomography. OR-PAM forms images by raster-scanning a tightly focused excitation spot, with lateral resolution set primarily by the optical focus (micron-scale) and axial resolution by the detector bandwidth [38, 49]. This imposes strong demands on beam quality and pointing stability: a near-diffraction-limited beam (or effective spatial filtering and single-mode delivery) is needed to maintain a tight, repeatable focus across the scan and to avoid resolution loss and artefacts [41, 49, 59].

OR-PAM builds an image point-by-point, so PRF is a primary driver of imaging speed. The shallow imaging depth shortens the acoustic time-of-flight window, so PRF can be pushed to the 10 s of kHz and beyond, and MHz-class A-line rates have been demonstrated for wide-field, high-speed scanning [59, 62]. At the same time, the tight focus results in high local fluence, so per-pulse energy must remain low to respect the MPE in the focused case, shifting many OR-PAM implementations toward nJ to low-$\mu$J pulses and relying on high PRF (and averaging when needed) rather than high per-pulse energy [49, 51, 52]. For quantitative OR-PAM and functional extensions, low pulse-to-pulse energy noise, precise triggering, and (when needed) fast wavelength switching are important to prevent striping and bias during scans and multispectral measurements [49, 65].

OR-PAM has most commonly been driven by nanosecond, high-beam-quality visible sources that can be tightly focused, historically using DPSS-pumped dye lasers to access haemoglobin-sensitive wavelengths, often combined with spatial filtering or single-mode delivery to preserve near-diffraction-limited focusing [38, 59]. High-speed OR-PAM has also been demonstrated with high-repetition-rate nanosecond fibre lasers to increase A-line rates while keeping per-pulse energy low at the focus [58]. To reduce size and cost, researchers have demonstrated laser-diode-based OR-PAM, which trades longer pulse widths for compactness and simpler system integration [145]. At the fastest end of functional OR-PAM, custom dual-wavelength, MHz-class pulsed lasers with rapid switching have been integrated for rapid sO$ _2$ mapping [62].

AR-PAM relaxes optical focusing requirements and determines lateral resolution through the acoustic aperture, typically in the 30–100 $\mu$m range [146, 147]. Acoustic resolution allows the use of lower beam quality illumination, supports higher pulse energies, and more readily accommodates fibre-coupled delivery, while providing greater penetration depth. Nanosecond-pulse DPSS lasers operated at a PRF of several to tens of kHz are commonly employed as a light source, particularly at a wavelength of 532 nm [148]. An OPO [149], dye laser [150], or SRS stage can provide multispectral imaging ability, all with some limitations for fast scanning: OPOs and dye lasers cannot be tuned at microsecond time scales, and SRS provides fixed wavelengths only.

#### 3.2.3 Photoacoustic intravascular and endoscopic imaging

PA imaging from minimally invasive probes has been investigated mostly for the arteries [151] and the gastrointestinal tract [152]. In endoscopes with a single acoustic transducer, every laser pulse yields one image line, and the image is formed by scanning the probe; in that sense, the scan sequence is similar to PA microscopy. Light is delivered through an optical fibre, which can be either of a step-index multi-mode fibre (MMF), a graded-index MMF [153], or a single-mode fibre (SMF). Fibre coupling to the appropriate fibre type is thus a crucial requirement for light sources to be used for endoscopy imaging. In most cases, endoscopic PA is a form of AR-PAM: images are accumulated by scanning a PA scan head across the tissue, delivering an unfocused or moderately focused beam to scattering tissue and receiving the signal in the narrow acoustic receive field of the transducer. Optical-resolution endoscopy systems were demonstrated using SMF-based probes, incorporating suitable focusing optics [154–156]. Acoustic-resolution intravascular and endoscopic PA systems can interrogate tissue over several millimetres, depending on wavelength, fluence, acoustic detection bandwidth, and probe geometry, whereas optical-resolution endoscopic implementations are typically confined to more superficial structures because of the short optical Rayleigh length and the need for precise probe positioning within the lumen.

Endoscopic and intravascular imaging poses a specific set of requirements: high PRFs are necessary to achieve frame rates compatible with real-time guidance and to mitigate motion artefacts. For real-time imaging of macroscopic tissue volumes and adequate sampling, a PRF of several kHz or higher is required [66, 157]. Good beam quality is required for efficient fibre coupling, where probe flexibility and size favour small fibre diameters [153, 158]. In addition, systems that target clinical use will need to be robust and compact. Endoscopic imaging is performed at close range, with a small laser spot. As a result, the MPE restricts the maximum pulse energy to low values, in the order of 10 s of microjoules or smaller.

Most studies targeting fast imaging ($&gt;$10 frames per second) have used DPSS lasers [159], often with wavelength conversion [66, 151]. DPSS microchip lasers offer a compelling trade-off between peak power and miniaturization, enabling integration into portable consoles [41]. Fibre lasers may emerge as a useful alternative in the future. For IVPA imaging of lipids (targeting 1210 nm or 1720 nm), nanosecond pulsed fibre lasers, and SRS sources have emerged as potentially suitable candidates [104, 105, 112]. These sources provide the necessary combination of high PRF (up to MHz), excellent beam quality ($M^2 < 1.2$) for efficient fibre coupling, and a compact, stable architecture.

**Figure 5.**
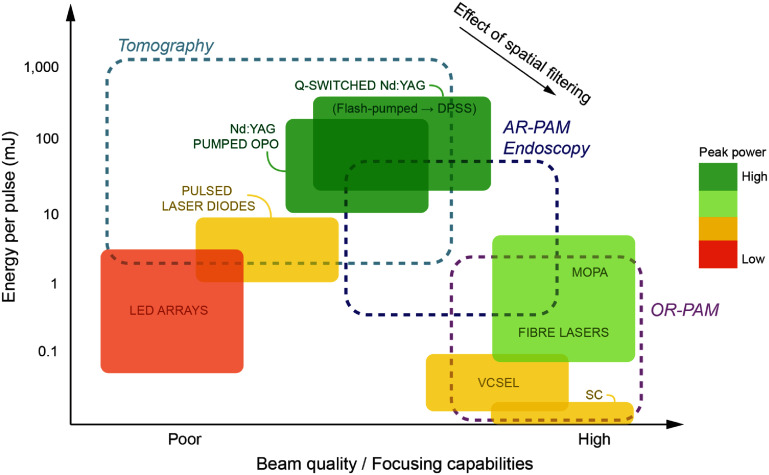
Comparison of commonly used photoacoustic excitation light-source classes (solid boxes) in the energy per pulse versus beam quality (focusing capability) space. Dashed envelopes delineate typical photoacoustic imaging regimes, including tomography, acoustic-resolution microscopy, endoscopic implementations, and optical-resolution microscopy. Colour represents the qualitative peak power associated with each source, which is intimately related to pulse duration, with longer pulses resulting in lower peak power (e.g. LED arrays). Higher beam quality can be achieved through spatial filtering of the beam or fibre coupling, at the expense of pulse energy.

Figure 5 compares the characteristics of various available light sources for PA imaging with the requirements of the different imaging regimes. This comparison highlights both areas of overlap, where multiple technologies may satisfy a given application, and gaps, most notably within the AR-PAM regime, where simultaneously achieving moderate pulse energy and high beam quality remains challenging, except through spatial filtering or lossy fibre coupling. Finally, a comparison between figure 5 and table 1 illustrates the general trend that excitation sources occupying the upper-right region of the diagram (higher pulse energy and superior beam quality) are typically associated with increased system complexity and cost.

### 3.3 Practical considerations for clinical implementation

Clinical PA imaging has been performed in specialized trials. These studies have generally been performed with imaging systems that are directly derived from research setups. Actual real-world application of PA imaging in clinical settings presents a set of requirements that are more stringent than in a research lab. In translating PA to clinical use, researchers and engineers need to design their imaging systems to comply with constraints in cost, usability, energy efficiency, safety, and other factors. Several of these have consequences for the choice of light source.

The main barrier to widespread clinical adoption of photoacoustic imaging is probably cost. OPO lasers commonly used for tomographic imaging are generally priced upwards of k$\unicode{x20AC}$100, similar to a complete high-end ultrasound system [3]. This aspect pushes the cost of combined PA/US platforms based on tunable solid-state lasers to several times that of advanced ultrasound-only systems. Although single-wavelength solid-state lasers are less expensive (thousands to tens of thousands of euros), they still represent a substantial added cost within an ultrasound system. LED-bars [10, 94], diode lasers [144, 160], and VCSELs [99, 101] that are suitable for tomographic imaging have the potential to become more competitive in price. LEDs and short-pulse diode lasers are available at a number of useful wavelengths at a similar cost to single-wavelength solid-state lasers, but greater volumes might make them more economical in the future. VCSELs can potentially be much cheaper (few hundred euro range), but presently are only available in a small number of wavelengths.

Some lasers need maintenance for stable operation. While semiconductor sources can be considered maintenance-free, pulsed solid-state lasers need regular service. Flashlamp-pumped systems require a lamp change every 10–20 million shots, typically, which adds to cost and disrupts operations. In addition, lasers with a free-space optical path may need occasional realignment and cleaning or replacement of optics. Both these interventions need to be performed by a trained engineer. For fibre-coupled systems, maintaining clean fibre ends is critical for reliable operation since the high pulse energies can cause damage. The operating lifetime of diode lasers, including the pump diodes in DPSS and fibre laser systems, is usually estimated at 50 000–100 000 hours of operation.

Other practical considerations in the choice of light source for clinical PA imaging relate to the fit of a laser system into the clinical environment. First, if high-power lasers are operated, both the patient and the physician must wear laser goggles. Access to the examination room must be controlled to prevent accidental exposure to bystanders. Second, the energy efficiency of different laser systems is highly variable. OPO lasers that deliver around 1 W of light to tissue may require a cooling capacity exceeding 1 kW. The examination room must be equipped to dissipate this heat. Third, these powerful water-to-air heat exchangers can produce a sound level of up to 65 dB(A), which interferes with conversation. Fourth, a high-power solid-state laser, with its associated power supply and chiller, is physically large and cumbersome to move around. Not every echography room will be able to accommodate one. Finally, PA excitation light needs to be delivered to the tissue under examination. The fibre bundles conventionally applied to achieve this can be heavy and affect the manoeuvrability of handheld probes.

Diode lasers and LEDs are more efficient and smaller, avoiding most of these impacts on practical usability. Diode lasers have been incorporated in handheld ultrasound probes [86], and LEDs are used in a holder mounted on the probe [10]. Excess heat dissipation through passive heat exchangers can cause the probe to heat up. While fibre coupling is not feasible for LEDs, diode lasers can potentially be delivered by an optical fibre, using suitable coupling optics. Diode lasers still require eye protection, and their high pulse frequency in combination with moderate pulse energy means that MPE limits must be carefully observed.

From a health economics perspective, the additional cost and practical burden of PA imaging compared to echo alone need to be offset by improved health outcomes. These can be quantified in well-defined clinical scenarios, favouring the development of imaging systems optimized for specific applications. The optimization determines system parameters such as pulse energy, speed, and a limited set of specific wavelengths. In such cases, the use of commercially available lasers at usable but suboptimal wavelengths may strike a working compromise between contrast and cost. Presently, lacking a large number of clear applications where PA imaging will improve outcomes, the expense of more versatile high-power tunable systems can present a challenge for commercialization.

Introduction of new technologies in a clinical setting is subject to regulations, which differ between countries. Regarding requirements of the light source, the US require compliance with the ANSI Z136 family of standards for laser safety, while European and major Asian jurisdictions (including Japan and China) use national regulations that are directly based on the IEC 60 825 series of standards. The general documents Z136.1 and IEC 60 825–1 define laser classes based on exposure hazards to eyes and skin. Furthermore, they describe the use of personal protective equipment, safety measures, requirements of enclosures, warning signs, and labels. The norms also include the MPE that we highlighted previously. The use of lasers in healthcare is specifically documented in ANSI Z136.3 and IEC 60 825–8.

Documentation required for a medical device marketing authorization generally requires a description of how the device complies with these and other applicable norms. For an imaging system incorporating a Class 4 laser, this includes the use of the wavelength- and optical-density-appropriate eye protection, a system preventing accidental exposure (e.g. an interlock system that switches the laser emission off when a door is opened), measures that ensure that the laser cannot run unattended (e.g. by including a foot pedal that needs to be pressed to operate it), training of personnel, warning signs, and many others. Most of these requirements also apply in a research setting.

### 3.4 Light source selection guide

**Figure 6.**
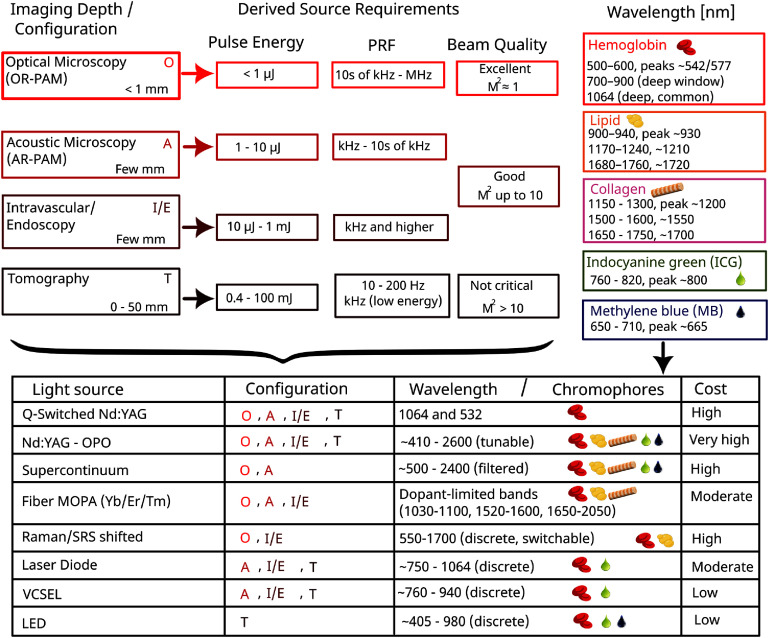
Simplified guide to light source selection for photoacoustic imaging.

Translating the trade-offs discussed in the previous sections into a practical selection pathway is highly desirable. Figure 6 presents a decision flow chart that guides the reader from application requirements to a shortlist of suitable excitation sources by progressively applying key constraints, including the target absorber and wavelength range, the required imaging depth, the resulting pulse energy and repetition frequency, and beam quality and fibre-coupling requirements. The intent is not to prescribe a single ‘best’ source, but to provide a guide to narrow the options and make the design choices and compromises explicit for a given photoacoustic modality.

## 4 Current gaps and future directions

Photoacoustic imaging is a potentially very powerful diagnostic modality with promising results in clinical studies. For clinical and commercial success of photoacoustic imaging, robust, compact, energy-efficient, cost-effective light sources at useful wavelengths are indispensable. Those wavelengths are prescribed by the molecules of interest and the optics of the tissue in which they are embedded. Multispectral imaging is essential for many applications. Furthermore, the pulses need to be short but powerful, at a repetition frequency that ideally allows averaging while maintaining a high frame rate, but avoids aliasing of subsequent pulses due to acoustic delay.

*Scientific applications:* For scientific research, where cost and maintenance are less of a concern, Nd:YAG-pumped OPOs meet these requirements for most applications. Their main limitation in some scenarios is their PRF. Studies requiring a PRF greater than about 100 Hz, such as microscopy (section 3.2.2), have typically sacrificed tunability. Fast, rapidly tunable lasers with a high-beam quality, a broad spectral reach, and greater narrow-band pulse energy than SC lasers, would enable PAM with more wavelength agility.

Quantitative PA flow imaging is another area that would benefit from wide-band tunable, high PRF lasers. Most flow imaging techniques work by tracking particle transit time in the imaging correlation volume, which can be either optically (OR-PAM) or acoustically bounded (AR-PAM, tomography) [161–164]. The autocorrelation decay time of the signal, or time shift between pulses with a controlled time difference, can be converted into particle velocity [165]. The accessible range of flow speeds depends on the delay between the pulses, and for physiological flows can be between 10 s of $\mu$s and 10 s of ms [166].

Lasers built for particle image velocimetry (PIV) offer a combination of high-power pulses and precisely controlled timing in pulse pairs or trains. PIV lasers typically work at wavelengths of 532 or 527 nm and have high beam quality, albeit at high cost. In PA flow imaging, dual Q-switched laser systems have been synchronized to achieve the same result [166, 167]. Fixed-wavelength sources like these do not allow for spectroscopic measurements. Combined flow and oxygenation measurements have been achieved with Raman-shifted lasers [69], which provide multiple fixed wavelengths.

*Clinical imaging:* Lasers for practically usable PA systems, applied in clinical settings, need to be robust, stable, compact, energy efficient, maintenance-free, and affordable. Semiconductor and fibre sources emerge as promising candidates. Diode lasers and VCSELs offer a range of wavelengths in the visible and NIR spectral regions, although wavelengths $\gt~\!\!\!\!\mu$m are scarce (1.0–1.06, 1.3, 1.55, and around 2.0 $\mu$m sources exist). This means that endogenous chromophores like lipids and collagen are not yet readily imaged with currently available high-pulse-energy semiconductor sources. High pulse energy diode lasers at wavelengths of 1.2 or 1.7 $\mu$m would enable lipid imaging [168], while collagen can be detected with wavelengths between 1.45 and 1.55 $\mu$m [139], carefully chosen to discriminate from a strong signal generated by water absorption.

High pulse energies in diode lasers can be accomplished by multi-junction structures such as bars and stacks. Because of the multi-emitter configuration, it is challenging to couple their output to optical fibres. Dedicated micro-optics may assist in conditioning the light to achieve efficient fibre coupling. Furthermore, a relatively long pulse duration is needed to achieve sufficient pulse energy, which means sensitivity of the PA signal to small features is limited. Shorter pulses require higher gain media or dedicated custom current drivers. For all of these developments, it is likely that the economy of scale may drive down cost. For this reason, it is interesting to explore sources that are developed for intrinsic high-volume applications such as the automotive industry, which uses pulsed infrared lasers for LIDAR, or consumer electronics.

High-power pulsed nanosecond fibre lasers are commercially available at wavelengths in the Yb$ ^{3+}$ and Er$ ^{3+}$ gain bands. Other fibre gain media, such as Tm-doped fibre, have been investigated to extend the spectral coverage to longer wavelengths [105, 169]. Specific wavelengths for lipid, collagen or water imaging can be created using SRS fibre amplifiers [112, 115]. These sources are highly suited for OR-PAM applications, with typical pulse energies of 100 s of nJ and PRFs of several 100 kHz.

AR-PAM or endoscopy systems have a greater viewing depth, and the acoustic delay can be of the order of 10 $\mu$s, necessitating lower PRF, especially if multispectral imaging or interleaved pulse-echo acquisition is desired. There is a need for lower PRF fibre lasers, but realizing these can be challenging because of amplified spontaneous emission build-up in CW-pumped amplifier stages. Synchronous pumping can remedy this concern but requires fibre lengths exactly matched to the desired PRF, which is more complex to build and sacrifices some flexibility.

Additionally, AR-PAM and endoscopy systems require a larger pulse energy than OR-PAM systems. Such pulses combine high energy ($ \gt\!\!10\mu$J) and short pulse duration ($\sim$10 ns) in a small fibre core (mode field diameter of a few $\mu$m), leading to strongly non-linear behaviour that is difficult to control. The use of large mode area fibres can reduce nonlinearity but also make the SRS generation less effective. Especially if such fibres need to be polarization-maintaining, or are part of the amplifier stage, doped with active ions (Er$ ^{3+}$ and/or Yb$ ^{3+}$), these are speciality items that require custom, thus expensive, fibre draws.

However, overall, the flexibility of fibre laser design, with many adjustable parameters, cavity and amplifier configurations, appears like a fertile area for the development of new lasers for PA applications that are presently lacking robust and efficient light sources, such as lipid and collagen imaging. Because of the complexity and nonlinearity of SRS MOPAs, it is also a field of scientific and engineering interest.

## 5 Conclusions

This review surveyed the parameter space of light sources for pulsed PA imaging, available light source technologies, and how they relate to the needs of different PA imaging implementations. Observing the MPE as an upper limit for the energy that can be applied to tissue in PA imaging, we have explored the relation between the system parameters, pulse energy, PRF and exposure time (for averaging) on one hand, and the resulting SNR gain on the other. We derived a relation between the PRF and exposure time that maximizes the SNR given a certain energy budget.

An array of fixed-wavelength or tunable, low-, medium- or high-PRF sources with different pulse energies was discussed in the context of various PA imaging applications. Tomographic imaging, where every laser pulse yields an image or a volume, favours a high pulse energy, low- to medium PRF source. OR-PAM, AR-PAM and endoscopy usually rely on point scanning to compose an image, and each laser pulse yields an image line, or even a point in OR-PAM. These forms of PA imaging require higher PRFs (tens of kHz to MHz) and lower pulse energies to comply with the MPE. The PRF is limited by the acoustic delay of the signal per acquisition.

Each of these applications may benefit from wavelength agility. Biological chromophores such as Hb/HbO$ _2$, lipids, collagen and melanin have absorption features that cannot always be probed by wavelengths available from common pulsed lasers. Hence, wavelength conversion techniques are important for the development of most PA imaging applications, and include both fixed energy shift methods such as SRS, and tunable ones like parametric generation.

We concluded with a discussion of conditions for successful clinical deployment, beyond the physical characteristics of the light source and imaging system. Cost-effectiveness, robustness, energy efficiency and user-friendliness are important in real-world use, and differentiate between various source technologies in addition to their physical characteristics. The combination of scientific novelty, clinical interest, economic considerations and what is physically possible will shape the future of PA imaging, served by innovative light sources that present a worthwhile research challenge in the coming decade.

## Data Availability

The data that support the findings of this study are openly available at the following URL/DOI: https://github.com/CBORT-NCBIB/MPE-Calculator-Skin [170].
